# Comment related to the publication “Elevation of transaminases after MMP® session with methotrexate for alopecia areata treatment ‒ How much do we know about the risks of systemic absorption of the technique?” ‒ Answer^⋆^

**DOI:** 10.1016/j.abd.2023.08.001

**Published:** 2023-09-23

**Authors:** Renata Heck, Renan Rangel Bonamigo

**Affiliations:** a Service of Dermatology, Hospital de Clínicas de Porto Alegre, Porto Alegre, RS, Brazil; b Faculty of Medicine, Universidade Federal do Rio Grande do Sul, Porto Alegre, RS, Brazil

*Dear Editor,*

We agree with the consideration that, regarding the cause of the increase in transaminase levels in the described patient, there are no means of affirming with certainty the association with the MMP® technique.^1^ We reinforce, however, that she had already shown an elevation of transaminase levels with the previous use of the medication when administered orally. We attributed it to the technique used after excluding other possible causes such as infections, alcohol consumption, physical activity, or use of any other medication, in addition to the transitoriness of transaminitis in relation to the procedure.

The technique involves the penetration of needles into the dermis,^2^, ^3^ where vascularization is prominent in the scalp, with biological plausibility in affirming the possibility of absorption of the drug used. Additionally the patient is likely to be susceptible to methotrexate-related adverse effects, despite the small, absorbed dose.

We reaffirm the importance of reporting unexpected adverse effects related to the recent use of percutaneous treatment techniques, aiming at patient safety through adequate monitoring.

## Financial support

None declared.

## Authors' contributions

Renan Rangel Bonamigo: Drafting and editing of the manuscript and critical review of important intellectual content; effective participation in research orientation; intellectual participation in the propaedeutic and/or therapeutic conduct of the studied cases; critical review of the literature; approval of the final version of the manuscript.

Renata Heck: Design and planning of the study; drafting and editing of the manuscript and critical review of important intellectual content; effective participation in research orientation; intellectual participation in the propaedeutic and/or therapeutic conduct of the studied cases; critical review of the literature; approval of the final version of the manuscript.

## Conflicts of interest

None declared.
